# Clinical utility of androgen receptor gene aberrations in circulating cell-free DNA as a biomarker for treatment of castration-resistant prostate cancer

**DOI:** 10.1038/s41598-019-40719-y

**Published:** 2019-03-11

**Authors:** Takayuki Sumiyoshi, Kei Mizuno, Toshinari Yamasaki, Yu Miyazaki, Yuki Makino, Kosuke Okasho, Xin Li, Noriaki Utsunomiya, Takayuki Goto, Takashi Kobayashi, Naoki Terada, Takahiro Inoue, Tomomi Kamba, Akihiro Fujimoto, Osamu Ogawa, Shusuke Akamatsu

**Affiliations:** 1 Department of Urology, Kyoto University Graduate School of Medicine, Kyoto, Japan; 2 Department of Drug Discovery Medicine, Kyoto University Graduate School of Medicine, Kyoto, Japan; 3 Department of Urology, Miyazaki University, Miyazaki, Japan; 4 Department of Urology, Graduate School of Medical Sciences, Kumamoto University, Kumamoto, Japan

## Abstract

The therapeutic landscape of castration-resistant prostate cancer (CRPC) has rapidly expanded. There is a need to develop noninvasive biomarkers to guide treatment. We established a highly sensitive method for analyzing androgen receptor gene (*AR*) copy numbers (CN) and mutations in plasma circulating cell-free DNA (cfDNA) and evaluated the *AR* statuses of patients with CRPC. *AR* amplification was detectable in VCaP cell line (*AR* amplified) genomic DNA (gDNA) diluted to 1.0% by digital PCR (dPCR). *AR* mutation were detectable in LNCaP cell line (*AR* T878A mutated) gDNA diluted to 0.1% and 1.0% by dPCR and target sequencing, respectively. Next, we analyzed *AR* status in cfDNA from 102 patients. *AR* amplification and mutations were detected in 47 and 25 patients, respectively. As a biomarker, *AR* aberrations in pretreatment cfDNA were associated with poor response to abiraterone, but not enzalutamide. In serial cfDNA analysis from 41 patients, most *AR* aberrations at baseline diminished with effective treatments, whereas in some patients with disease progression, *AR* amplification or mutations emerged. The analysis of *AR* in cfDNA is feasible and informative procedure for treating patients with CRPC. cfDNA may become a useful biomarker for precision medicine in CRPC.

## Introduction

Prostate cancer (PCa) is the second most frequently diagnosed cancer among males worldwide. PCa is driven by androgen receptor (AR) signaling, with the standard treatment for advanced disease being androgen deprivation therapy. However, most patients eventually gain resistance and develop castration-resistant prostate cancer (CRPC). The primary mechanism underlying CRPC is reactivation of the AR pathway. This includes *de novo* androgen synthesis by cancer cells, AR gene (*AR*) amplification and mutations, and generation of truncated splice variants lacking the ligand binding domain (LBD)^1,2^. Potent AR axis-targeted agents (ARATs) such as abiraterone and enzalutamide prolong survival of patients with CRPC; however, nearly a third of the patients show primary resistance to ARATs^3,4^. Taxanes such as docetaxel and cabazitaxel are also known to prolong survival of patients with CRPC. In addition, multiple AR targeting and non-AR targeting agents are currently in development. Given the wide selection of treatments for CRPC, it is important to optimize the treatment sequence and promote precision medicine based on molecular and genetic biomarkers.

In the past 5 years, several large studies have identified recurrent somatic mutations, copy number alterations, and chromosomal rearrangements from CRPC metastatic sites^5–7^. However, it is difficult to routinely obtain tissue in PCa, which predominantly metastasizes to bone. Even if successfully collected, the sample may contain low tumor volume and not amenable to genetic studies. Furthermore, CRPC is a highly heterogeneous disease, and biopsy of a single site may not reflect the molecular characteristics of all tumor sites. As an alternative to tissue biopsy, there is a growing interest in liquid biopsies such as circulating tumor cell (CTC) and circulating cell-free DNA (cfDNA). In PCa, several groups have recently shown the association of *AR* amplification and mutations in plasma cfDNA with poor responses to abiraterone and enzalutamide in patients with CRPC^8–12^. However, predictive values of these aberrations remain controversial^13^. Moreover, there is still a technical challenge to analyze highly fragmented (150–200 bp) and diluted (nanograms per 1 ml plasma) cfDNA and reliably detect those shed from tumor cells (circulating tumor DNA; ctDNA) which generally comprises less than 10% of cfDNA. Herein, we established a robust method to analyze the *AR* copy number (CN) and mutations in plasma cfDNA by combined use of digital PCR (dPCR) and multiplex PCR based target sequencing. We analyzed the *AR* status of Japanese patients with CRPC to evaluate the utility of cfDNA as a novel biomarker.

## Results

### The development of *AR* CN and mutations analysis in cfDNA

**Figure 1:**
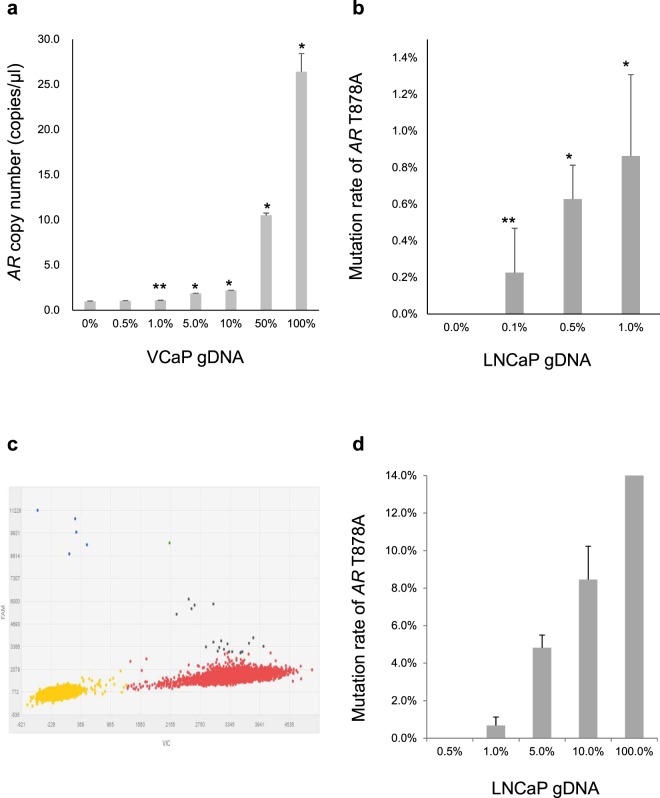
The sensitivity of copy number (CN) and mutation analysis by digital PCR (dPCR) and target sequencing. (**a**) LNCaP (*AR* CN neutral) genomic DNA (gDNA) was spiked with VCaP (*AR* amplified) gDNA in serial dilution. *AR* amplification could be detected by dPCR even when VCaP gDNA was diluted to 1.0%. Stars (p < 0.01* and p = 0.015**) indicate that *AR* CN in diluted VCaP gDNA are significantly higher than that in LNCaP gDNA by the Student’s t-test (n = 3). (**b**) gDNA from healthy males (*AR* wild type) was spiked with LNCaP (*AR* T878A mutated) gDNA in serial dilution. The *AR* T878A mutation could be detected at 0.1% dilution by dPCR. Stars (p < 0.01* and p = 0.014**) indicate that the mutation rates in diluted LNCaP gDNA were significantly higher than those observed in gDNA from healthy males by the Student’s t-test (n = 3). (**c**) Scatter plots of dPCR analysis for the gDNA from healthy males spiked with 0.1% LNCaP gDNA. The mutation detection rate was 0.09%. The blue dots show positive droplets for the *AR* T878A mutation. The red dots show positive droplets for the *AR* wild type. The yellow dots show empty droplets. (**d**) *AR* target sequencing for gDNA from healthy males spiked with LNCaP gDNA in serial dilution. *AR* T878A mutation could be detected up to 1.0% dilution. All error bars indicate standard deviation.

*AR* CN analysis by dPCR was first tested using VCaP (*AR* amplified) and LNCaP (*AR* CN neutral) cell lines. *AR* CN in VCaP genomic DNA (gDNA) and LNCaP gDNA were 25.02 copies/µl and 0.94 copies/µl, respectively (Supplementary Fig. S1a). In serial dilution, *AR* amplification could be detected when VCaP gDNA was diluted by LNCaP gDNA to 1.0% (Fig. 1a). In *vivo*, *AR* amplification could be detected in plasma cfDNA from mice implanted with VCaP. In comparison, *AR* CN in cfDNA from mice implanted with LNCaP was 1.07 copies/µl, which was not statistically different from that in LNCaP gDNA (p = 0.064). These results confirmed the establishment of a successful assay for CN analysis in cfDNA (Supplementary Fig. S1b).

Similarly, *AR* mutations analysis by dPCR was tested using cell lines. T878A could be detected in LNCaP (*AR* T878A mutated) gDNA and cfDNA from mice implanted with LNCaP, but not in VCaP (*AR* wild type) (Supplementary Fig. S2a–d). In serial dilution, T878A could be detected even when LNCaP gDNA was diluted to 0.1% (Fig. 1b,c). By target sequencing, T878A could be detected when LNCaP gDNA was diluted to 1.0%, but not at 0.5% (Fig. 1d). In the range of 0.5% to 1.0%, other mutation candidates that have not been reported previously in LNCaP were detected (Supplementary Table S1). However, these candidates were undetectable when a different DNA polymerase was used for library preparation. Mutation candidates in the range of 0.5% to 1.0% were also detected in cfDNA from 3 healthy males, two of which corresponded to the variants that became undetectable in LNCaP after changing DNA polymerase (Supplementary Table S2), indicating false positive detections. Therefore, for mutation analysis by target sequencing in human cfDNA samples, we set a cut-off of variant allele frequency (VAF) of 1.0%. However, for the mutations in known hot spots (L702H, W742C, W742L, H875Y and T878A), we set the cut-off at 0.5%, and validated the mutations with dPCR.

### The evaluation of *AR* status in plasma cfDNA from patients with CRPC

One hundred two patients with CRPC were recruited, and a total of 147 blood samples were collected (45 were collected serially during treatment) (Supplementary Fig. S3). Of 102 baseline cfDNA samples, 83 were collected upon biochemical or clinical progression to ongoing treatment, and 19 were collected during response to treatment. Baseline patient characteristics are shown in Table 1. Notably, many patients had histories of treatments with various conventional anti-androgens and estrogens since the majority of them were already castration-resistant before abiraterone and enzalutamide became widely available in Japan. The median cfDNA concentration from patients with CRPC [11.36 ng/ml (range: 2.84–1464)] was significantly higher than that from healthy males [5.52 ng/ml (range: 3.02–12.96)] (p < 0.01) (Supplementary Fig. S4).

**Table 1:** Patients’ Characteristics at baseline

	All patients (n = 102)
Age, median (range), years	74 (48–96)
PSA, median (range), ng/ml	15.8 (0.008–2082)
Gleason score, No. (%)	
6–7	18 (17.6)
8–10	80 (78.4)
Unknown	4 (3.9)
Metastasis, No. (%)	
Yes	94 (92.2)
No	8 (7.8)
Site of Metastasis, No. (%)	
Lymph node	58 (56.9)
Bone	80 (78.4)
Lung	16 (15.7)
Liver	8 (7.8)
Other	6 (5.9)
ECOG PS, No. (%)	
0–1	73 (71.6)
≥2	29 (28.4)
Time from starting ADT, median (range), months	55.9 (4.1–207.6)
Time from CRPC diagnosis, median (range), months	24.0 (0–140.7)
Hemoglobin	
Median (range), g/dl	12.4 (6.6–15.7)
<LLN No. (%)	19 (18.6)
ALP	
Median (range), U/L	275.5 (78–3208)
≥360, No. (%)	32 (31.4)
LDH	
Median (range), U/L	204 (77–2280)
≥227, No. (%)	29 (28.4)
Treatment immediately prior to baseline sample collection, No. (%)	
Hormone therapy	
Bicalutamide	16 (15.7)
Flutamide	17 (16.7)
Estramustine phosphate	7 (6.9)
Abiraterone	20 (19.6)
Enzalutamide	21 (20.6)
Others	10 (9.8)
Chemotherapy	
Docetaxel	5 (4.9)
Cabazitaxel	4 (3.9)
Paclitaxel and Carboplatin	2 (2.0)
No. of resistance to anti-androgen therapy/chemotherapy, median (range)	3 (0–9)
Resistance to each treatment, No. (%)	
Hormone therapy	
Bicalutamide	99 (97.1)
Flutamide	65 (63.7)
Estramustine phosphate	35 (34.3)
Abiraterone	30 (29.4)
Enzalutamide	32 (31.4)
Others	23 (22.5)
Chemotherapy	
Docetaxel	27 (26.5)
Cabazitaxel	5 (4.9)
Paclitaxel and Carboplatin	3 (2.9)

Abbreviations: PSA, prostate-specific antigen; ECOG PS, Eastern Cooperative Group Performance Status; ADT, androgen deprivation therapy; CRPC, castration-resistant prostate cancer; ALP, Alkaline Phosphatase; LDH, Lactate dehydrogenase; LLN, Lower Limit of Normal.

**Figure 2:**
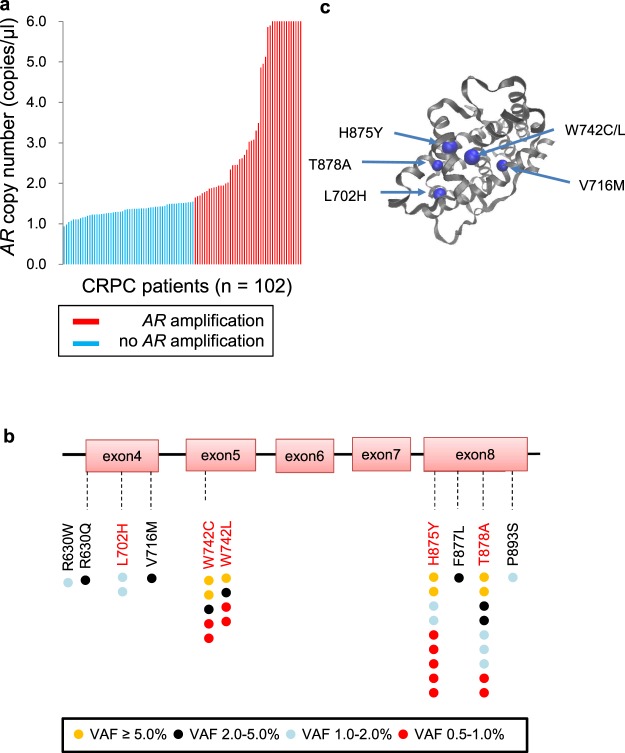
Landscapes of *AR* copy number (CN) and mutations in cfDNA from patients with CRPC (n = 102) at baseline. (**a**) *AR* amplification was defined as *AR* CN > 1.54 copies/μl based on *AR* CN in cfDNA from healthy males. The red lines show the cases with *AR* amplification, and the blue lines show the cases with no *AR* amplification. (**b**) The locations of the mutations identified are mapped on the *AR* gene. Each colored circle represents a single mutation in a single sample. The red letters indicate the known hotspots on ligand binding domain (LBD). (**c**) Structured 3D image of an AR protein showing that V716M is located in the proximity of the known hotspots in LBD. VAF, variant allele frequency.

*AR* CN in patients with CRPC ranged from 0.94 copies/µl to 165.8 copies/µl, and 47 of 102 (46.1%) patients had *AR* amplification at the time of baseline sample collection (Fig. 2a). Importantly, *AR* amplification was detected in 46 of the 83 (55.4%) patients who had baseline samples collected at disease progression, but in only 1 of the 19 (5.3%) patients whose samples were collected during the response to treatment. *AR* target sequencing in all cfDNA samples was successfully performed, and median coverage of each amplicon was >10000×. *AR* LBD mutations were identified in 25 of 102 (24.5%) patients (Fig. 2b). Nine of these 25 patients had 2 mutations, and each VAF ranged from 0.56% to 24.59%. The rate of *AR* mutation in samples collected at disease progression (21/83, 25.3%) was not significantly different from that sampled during response to treatment (4/19, 21.1%). Most of the mutations were located at known hot spots. The F877L mutation is reportedly associated with resistance to enzalutamide^14,15^ and was detected in one sample collected upon development of resistance to enzalutamide. Four additional mutations (R630W, R630Q, V716M and P893S) were identified. R630Q and V716M were identified at a relatively high VAF (3.25% and 3.64%, respectively). Importantly, we determined that V716M was located near the known LBD hot spots in the 3D structure of the AR protein, suggesting that this amino acid change might alter ligand binding of *AR* and associates with treatment resistance (Fig. 2c). R630Q and V716M were identified in cfDNA collected at the time of resistance to abiraterone and bicalutamide, respectively. Both patients subsequently responded to enzalutamide.

*AR* status in cfDNA was compared to matched CRPC tissue gDNA in 12 patients (Supplementary Table S3). In *AR* CN analysis, concordance was confirmed in 7 of 12 (58.3%) patients. All 5 patients with discordance showed *AR* amplification in gDNA from the prostate, but not in cfDNA. In *AR* mutation analysis, 10 of 12 (83.3%) patients showed completely concordant results. Patient KU-112 had W742L in cfDNA, but not in gDNA from the prostate, possibly reflecting tumor heterogeneity.

### The association between *AR* aberrations in cfDNA and clinicopathological factors

**Figure 3:**
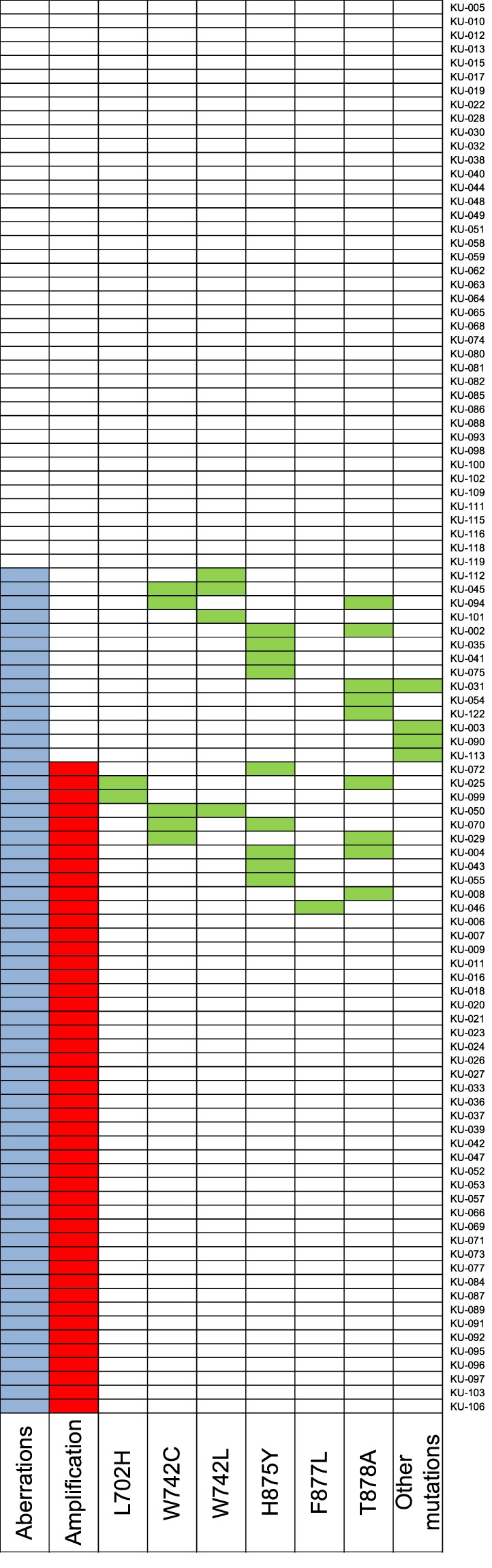
Integrated data of *AR* copy number and mutations in cfDNA from patients with CRPC (n = 102) at baseline. The longitudinally placed boxes indicate each sample. Sixty-one of 102 (59.8%) patients had *AR* aberrations (amplification and/or mutations) in cfDNA. Blue, red and green squares indicate *AR* aberrations, amplifications, and mutations, respectively.

The summary of *AR* status in cfDNA for each patient is presented in Fig. 3. Sixty-one of 102 (59.8%) patients had *AR* aberrations (amplification and/or mutations) in cfDNA. Eleven of these 61 patients had both *AR* amplification and mutations, which was inconsistent with other reports that indicated a tendency toward mutual exclusivity^6,11^. Clinicopathological factors associated with *AR* aberrations in cfDNA were analyzed in 83 patients who underwent blood collection at disease progression. Fifty-six of the 83 (67.5%) patients had *AR* aberrations, and we showed that Hb ≤ LLN, cfDNA concentration, increasing number of prior systemic treatments and resistance to estramustine phosphate, abiraterone, enzalutamide or docetaxel were significantly associated with the presence of *AR* aberrations in cfDNA by univariate analysis (Supplementary Table S4). Since only 8 patients were treated with chemotherapy immediately prior to baseline sample collection, there was no statistical difference in the frequency of *AR* aberrations between samples collected after hormone therapy progression and chemotherapy progression (Supplementary Table S4).

### The evaluation of *AR* aberrations in cfDNA as a biomarker of response to abiraterone and enzalutamide

**Figure 4:**
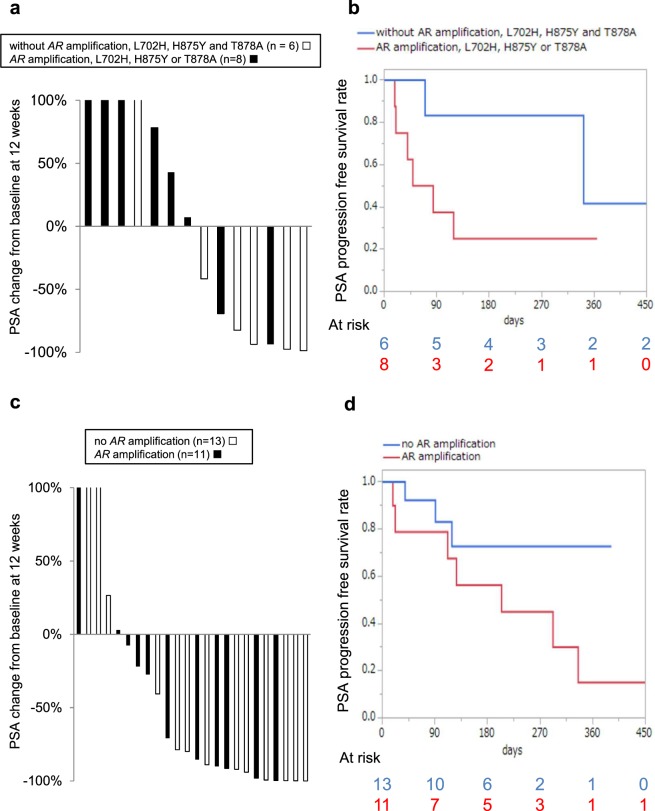
Clinical outcomes of abiraterone (n = 14) and enzalutamide (n = 24) therapy according to *AR* aberrations. (**a**) Waterfall plot of %PSA change from baseline at 12 weeks stratified by *AR* status for abiraterone. (**b**) Kaplan-Meier analysis of PSA-PFS in patients treated with abiraterone. Median PSA-PFS in the patients with *AR* amplification, L702H, H875Y or T878A in cfDNA tended to be shorter than those without these aberrations (median 66.5 days versus 342 days, p = 0.049 by Wilcoxon test and p = 0.087 by log-rank test). (**c**) Waterfall plot of %PSA change from baseline at 12 weeks stratified by *AR* status for enzalutamide. (**d**) Kaplan-Meier analysis of PSA-PFS in patients treated with enzalutamide. Median PSA-PFS was not significantly associated with *AR* status in cfDNA (median 205 days versus not reached, p = 0.212 by Wilcoxon test and p = 0.117 by log-rank test).

Of the 102 patients, 81 switched treatment after baseline cfDNA collection. Fourteen, 24 and 25 patients were initiating treatment with abiraterone, enzalutamide, and docetaxel/cabazitaxel, respectively. The clinical outcomes of each treatment were evaluated by the percentage of change in prostate-specific antigen (PSA) from baseline at 12 weeks (or earlier for those who discontinued treatment) and PSA progression-free survival (PSA-PFS) according to Prostate Cancer Working Group 2 Criteria^16^. For abiraterone, patients with *AR* mutations known to be associated with drug resistance (L702H, T878A or H875Y)^8,9^ or those with *AR* amplifications had poorer PSA response compared to patients without these aberrations (Fig. 4a). In the patients with *AR* CN neutral and mutations at other locations (R630W, W742C, W742L and P893S), PSA was reduced by more than 80.0%. Median PSA-PFS in the patients with these aberrations also tended to be shorter than those without the aberrations (median 66.5 days versus 342 days, p = 0.049 by Wilcoxon test and p = 0.087 by log-rank test) (Fig. 4b). On multivariable Cox proportional hazard analysis, *AR* aberrations was independently significantly associated with PSA-PFS (Supplementary Table S5). In contrast, in the enzalutamide group, PSA change and PSA-PFS in the patients with *AR* amplification were not significantly different from those without *AR* amplification (median 205 days versus not reached, p = 0.212 by Wilcoxon test and p = 0.117 by log-rank test) (Fig. 4c,d and Supplementary Table S6). In the docetaxel/cabazitaxel group, almost all patients had *AR* aberrations, and PSA change did not differ relative to *AR* status (Supplementary Fig. S5).

### Serial analysis of *AR* status in cfDNA

**Figure 5:**
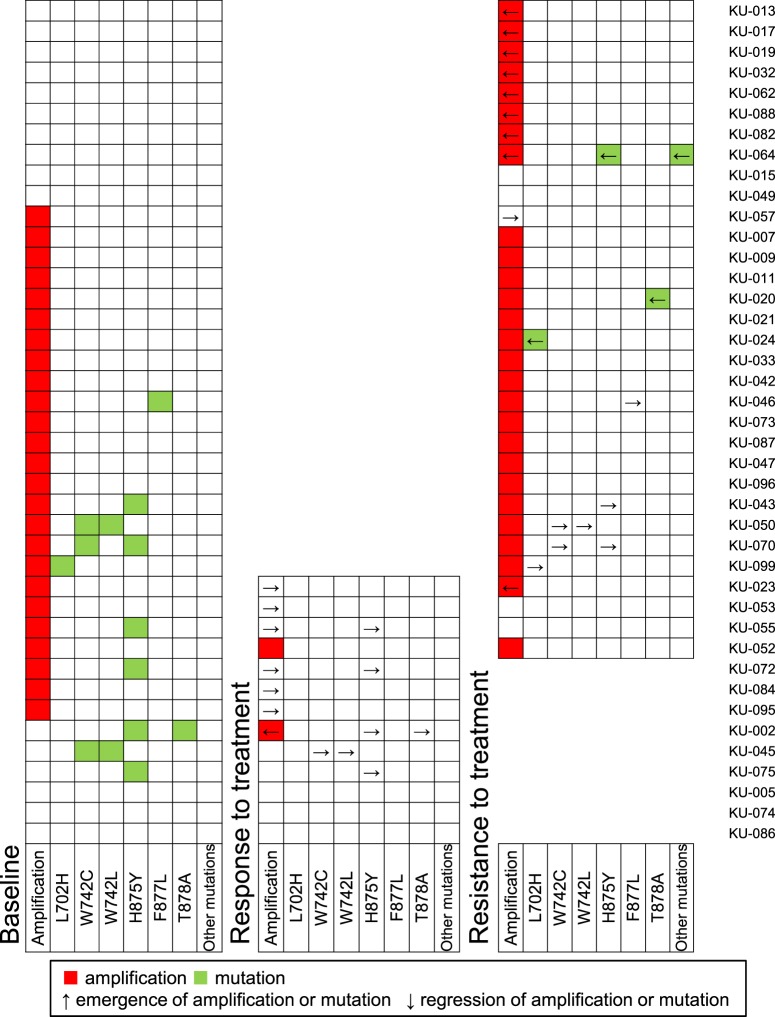
Integrative data of *AR* status in cfDNA at baseline, while responding to treatment and during treatment resistance. The longitudinally placed boxes indicate each sample. Red and green squares indicate *AR* amplification and mutations, respectively. Up and down arrows indicate emergence and regression of *AR* amplification or mutations, respectively.

In 41 patients, blood samples were collected multiple times during treatment to track *AR* status (Fig. 5). cfDNA samples from 37 patients were collected twice (at baseline and at the time of response or resistance to treatment), whereas 4 patients had cfDNA collected three times, at baseline, during response and at resistance to treatment. Thirteen of the 41 patients had cfDNA analyzed at baseline and during response to treatment. Among them, 7 patients had *AR* amplifications at baseline; however, amplification became undetectable in 6 patients during treatment with enzalutamide (n = 3), abiraterone (n = 1), docetaxel (n = 1) and cabazitaxel (n = 1). In one patient, *AR* amplification was newly detected at the time of response to treatment with paclitaxel and carboplatin. *AR* mutations were detected in 5 patients at baseline, all of which became undetectable during the patients’ responses to abiraterone (n = 2), enzalutamide (n = 1), docetaxel (n = 1) and paclitaxel and carboplatin (n = 1).

Of the 41 patients, we collected blood samples from 32 patients at baseline and at the time of resistance to treatment, including 4 patients who also had cfDNA analyzed while they were responding to therapy. Of the 22 patients with *AR* amplification at baseline, amplification remained detectable in 19 patients at the time of resistance, whereas *AR* amplification became undetectable in 3 patients (KU-053, KU-055 and KU-057). Among them, KU-053 and KU-055 were treated with enzalutamide after blood collection at baseline, and *AR* amplification became undetectable during their response, which remained undetectable at the time of progression. In patient KU-055, the *AR* mutation (H875Y), whose VAF at baseline was 21.3%, also became undetectable during treatment with enzalutamide and remained undetectable at progression. Intriguingly, this patient subsequently responded to abiraterone (Supplementary Fig. S6). Of the 10 patients without *AR* amplification at baseline, amplification was newly detected in 8 patients at the time of treatment resistance, suggesting that *AR* amplification arose as an adaptive response to treatment. In mutation analysis, all *AR* mutations detected at baseline became undetectable at progression. The patient with F877L (KU-046) was subsequently enrolled in a clinical trial for a vaccine therapy after exhibiting resistance to enzalutamide, and the mutation became undetectable upon progression to this therapy. T878A, L702H, H875Y, Q641R and D733N were newly detected in 3 patients (KU-020, KU-024 and KU-064) during treatment with abiratetone, docetaxel and estramustine phosphate, respectively.

## Discussion

*AR* amplification can drive CRPC progression and is present in up to 50% of CRPC tissues. CN analysis in cfDNA has been performed by several methods; array comparative genomic hybridization, next generation sequencing (NGS), real time PCR and dPCR^8–13,17^. In the present study, we showed that *AR* amplification could be detected by dPCR even when VCaP gDNA was diluted to 1.0%. The rate of *AR* amplification in cfDNA from patients with CRPC (46.1%) was comparable to that in other reports, suggesting that our method that used dPCR was a simple and robust approach to analyze CN in cfDNA. However, the concordance rate of *AR* amplification status between cfDNA and matched tumor tissue gDNA was only 58.3%. All 5 patients with discordant statuses had *AR* amplification detected in gDNA from prostate tissues, but not from matched cfDNA. Notably, all the patients with discordance only had low volume bone metastases, raising the possibility that low yield ctDNA was undetectable in these cases. In addition, *AR* CN in 2 patients (KU-112 and KU-116) was near the cut-off line for determining amplification and might have been falsely considered no amplification by our criteria. Increasing the sensitivity of dPCR technology may aid in detecting *AR* amplification in future cases.

*AR* mutations can also drive CRPC progression. Gain-of-function mutations such as L702H, W742C, W742L, H875Y and T878A can change ligand binding affinity, which results in increased sensitivity to steroid ligands or the conversion of anti-androgens to agonists^1,2^. *AR* mutations in cfDNA have been detected by NGS or dPCR^8,9,11–13^. NGS can evaluate targeted genes comprehensively; however, its sensitivity is restricted by the inherent detection limits of NGS technology which are around 1.0%. Digital PCR can detect rare mutations with a prevalence as low as 0.1%, however, it can evaluate only one mutation in one PCR reaction. In the present study, we combined 2 methods: initial screening of LBD mutations by multiplex PCR based target sequencing, followed by dPCR confirmation of hotspot mutations whose VAFs were between 0.5%-1.0%. By this approach, many hotspot mutations in the range of 0.5%-1.0% could be validated by dPCR. We did not validate other candidates outside of known hotspots; however, it is possible that these candidates also contain important biological information^18,19^. Therefore, efforts are underway to increase the sensitivity and specificity of NGS-based analysis using molecular barcodes whose utility have not yet been shown in cases of prostate cancer.

Interestingly, the number of patients with concomitant *AR* amplification and mutations in this study was higher than previously reported. In contrast to many studies in which analysis was performed after 1st or 2nd line treatments for CRPC, most of the patients in the present study had been exposed to multiple lines of AR axis-targeted treatments which contained anti-androgen therapies hardly used in Western countries (i.e. estramustine phosphate). Exposure to multiple agents targeting the AR pathway might have produced a generation of diverse clones with different mechanisms of resistance. In the present study, the number of prior systemic treatments and resistance to estramustine phosphate abiraterone, enzalutamide, or docetaxel were significantly associated with the presence of *AR* aberrations in cfDNA; however, resistance to bicalutamide and flutamide were not. This indicates that *AR* aberrations tend to accumulate more after resistance to second line hormonal therapy, compared to first line complete androgen blockade.

The utility of *AR* aberrations in cfDNA as predictors of responses to abiraterone and enzalutamide is still controversial. Initial studies have shown that *AR* aberrations in cfDNA are linked to poor outcomes with abiraterone and enzalutamide^8–12^, whereas a recent report showed that only strong *AR* gain (CN ≥ 8 copies) was associated with poor responses in patients unexposed to prior treatments for CRPC^13^. Despite our small sample size, we showed that *AR* amplification, L702H, T878A, or H875Y tended to be associated with poor response to abiraterone, and that *AR* aberrations were not associated with response to enzalutamide. The difference in outcomes between the 2 treatments is attributable to the different pharmacological mechanisms of each treatment; abiraterone mostly indirectly blocks the AR pathway via androgen biosynthesis, whereas enzalutamide blocks AR directly as an antagonist, and was developed to potently block the AR pathway within the context of AR overexpression^20,21^.

We serially analyzed cfDNA in 41 patients to evaluate dynamic changes in *AR* status during treatment. Almost all amplifications and mutations at baseline became undetectable during the response to treatment, possibly reflecting decreased tumor burden^11^. In some patients, *AR* amplification and mutations at baseline disappeared by the time of progression, possibly indicating that the clones that have gained resistance to previous treatments by acquiring *AR* aberrations were mostly eliminated by the subsequent treatments and became non-dominant clones at later progression. Quite intriguingly, in one patient (KU-055) with an *AR* amplification and H875Y at baseline, these aberrations became undetectable by the time of response to enzalutamide and did not re-emerge upon acquiring resistance. It is possible that tumor clones with the *AR* aberrations regressed dramatically by exposure to enzalutamide, and the tumor may have eventually acquired resistance to enzalutamide by other mechanisms such as increased de novo steroidogenesis through overexpression or mutation of *HSD3B1*^22^. The patient responded to subsequent treatment with abiraterone. Several studies have shown that response to abiraterone following enzalutamide is limited^23,24^; however, the present case raises the possibility that, on rare occasions, enzalutamide may eradicate the clones with *AR* aberrations associated with abiraterone resistance and re-sensitize the tumor to abiraterone. These observations, if further replicated in future studies, may lead to cfDNA based precision medicine in which optimal treatment sequence of CRPC could be guided by liquid biopsy.

This is the first report to evaluate *AR* status in cfDNA from Japanese patients with CRPC, but we stress that this study has several notable limitations. The sample size was small and the heterogeneous patient cohort exhibited a variety of baseline characteristics. Larger prospective studies are required. Since the current study focused on cfDNA, we did not analyze AR splice variants. In particular, AR-V7 in CTC has been reported to be a prognostic biomarker associated with resistance to abiraterone and enzalutamide^25^. AR overexpression including *AR* amplification or *AR* genomic structural rearrangements are known to be involved in the expression of AR splice variants^26^. In the future, it will be important to analyze CTC and cfDNA concurrently. Additionaly, a recent study performed targeted 72-gene sequencing of cfDNA in patients with CRPC and identified *BRCA2* and *TP53* mutations in cfDNA as predictive biomarkers associated with poor outcomes of abiraterone and enzalutamide^13^. Genomic aberrations other than *AR* also need to be evaluated within the context of a large cohort of patients with CRPC.

In conclusion, we have demonstrated that the analysis of *AR* status in cfDNA, obtained from a minimally invasive blood sample, is feasible and informative for patients with CRPC. *AR* aberrations in cfDNA have the potential to become useful biomarkers in patients with CRPC.

## Methods

### Patient cohort

One hundred two patients with CRPC and 15 healthy males were recruited at Kyoto university hospital between September 2015 and November 2017. Of the 15 healthy males, 6 underwent surgery for benign prostate hyperplasia before blood collection and were pathologically confirmed to be negative for PCa. The remaining 9 were healthy volunteers under the age of 40. All human experiments were approved by the ethical committees at Kyoto University Hospital (G1083). Written informed consent was obtained from all patients. All human experiments were performed in accordance with Japanese ethical guidelines for human genome/gene analysis research and ethical guidelines for medical and health research involving human subject.

### Blood collection and plasma preparation

A blood volume of 8.5–10 ml was collected in an EDTA-containing tube, Cell-Free DNA BCT (Streck) or Cell-Free DNA Collection Tube (Roche). Plasma was isolated by 2 step centrifugation (1600 g × 15 min and 4100 g × 10 min) within 2 h of blood collection for EDTA-containing tubes, or within 7 days for the other tubes. Plasma was stored at −80 °C until cfDNA extraction.

### cfDNA and gDNA extraction

cfDNA was extracted from 4–6 ml plasma using the QIAamp Circulating Nuclear Acid Kit (Qiagen) according to the manufacturer’s protocol. cfDNA concentration was measured using the Qubit 3.0 Fluorometer (ThermoFisher Scientific). Extracted DNA in the first several samples were run on the Bioanalyzer 2100 (Agilent Techonologies) to evaluate successful extraction of cfDNA. The DNA exhibited a peak at 168 bp which was consistent with that of cfDNA (Supplementary Fig. S7).

gDNA from WBC and tumor tissue was extracted using the DNeasy Blood and Tissue Kit (Qiagen) and the QIAamp DNA Mini Kit (Qiagen), respectively. gDNA concentration was measured using the Nanodrop 2000 spectrophotometer (ThermoFisher Scientific). cfDNA and gDNA were stored at −30 °C.

### *AR* CN analysis

*AR* CN was analyzed using the QuantStudio 3D Digital PCR system (ThermoFisher Scientific). PCR reaction was prepared with 7.5 μl of QuantStudio3D Digital PCR master mix (ThermoFisher Scientific), 0.75 μl of Taqman Copy Number Assay for *AR* (Assay ID: Hs04107225), 0.75 μl of Taqman Copy Number Reference assay for *RNaseP* (Assay ID: 4403326) and cfDNA or gDNA (about 5 ng) in a total volume of 15 μl. PCR reaction was loaded onto the QuantStudio 3D Digital PCR Chip (ThermoFisher Scientific) and amplified on ProFlex 2x Flat PCR System (ThermoFisher Scientific). The annealing and extension temperatures were set at 60 °C, and PCR was run for 39 cycles. After PCR amplification, chips were read on the QuantStudio 3D Digital PCR Instrument (ThermoFisher Scientific), and a secondary analysis was performed with QuanStudio 3D Analysis Suit Cloud software (ThermoFisher Scientific). *AR* CN was calculated using *RNaseP* as an internal control. The cut-off for indicating a positive *AR* amplification in cfDNA was *AR* CN > 1.54 copies/μl, which was the average plus 2 standard deviations of *AR* CN in cfDNA obtained from healthy males.

### *AR* mutations analysis

*AR* mutations were evaluated by 2 approaches: dPCR and target sequencing. To detect *AR* L702H, W742C, W742L, H875Y and T878A mutations by dPCR, validated Taqman SNP genotyping assays (Assays ID:C_356510059_10, C_175239651_10 and C_175239649_10) and custom-made Taqman SNP genotyping assays were used. The thermal cycling protocol was similar to that used in *AR* CN analysis; however, the annealing and extension temperatures and cycle number were changed to 56 °C and 39 cycles for L702H, W742C and W742L, 54 °C and 55 cycles for H875Y, and 62 °C and 39 cycles for T878A.

*AR* mutations analysis by target sequencing was based on multiplex PCR based deep sequencing^11^. In brief, a total of 11 primer sets spanning the *AR* LBD were designed and amplified by 3 sets of multiplex PCR which was designed to contain non-overlapping target regions (first PCR). KAPA HiFi HotStart ReadyMix (KAPA BIOSYSTEMS) and 2 ng of cfDNA were used per reaction. Successful amplification was confirmed by agarose gel electrophoresis, and the PCR products were purified by AMPure XP beads (Beckman coulter). Next, overhang adapters specifically designed for Illumina sequencing were attached to purified first PCR amplicons using primers that were used for first PCR with an overhang adapter sequence (second PCR). After purification, the concentration of each set of second PCR amplicons was measured using the Qubit 3.0 Fluorometer, and the amplicons were pooled by sample. Finally, a limited cycle amplification was performed to attach sample-specific barcode to second PCR amplicons using KAPA HiFi HotStart ReadyMix and the Nextera XT Index Kit (Illumina) (third PCR). Agarose gel electrophoresis, purification, and concentration measurement were performed, and the PCR products from 96 samples were pooled. Library quality and quantity were evaluated using the Bioanalyzer 2100 and qPCR according to the Illumina qPCR Quantification Protocol Guide, and the libraries were normalized. Sequencing was performed on the Illumina Miseq according to the manufacturer’s instructions. Sequencing run included serially diluted LNCaP gDNA as a positive control, and WBC gDNA from 13 patients with CRPC and cfDNA from healthy males as negative controls.

The Illumina Miseq generated raw images utilizing MiSeq Control Software v2.2 and base calling through an integrated primary analysis software called Real Time Analysis v1.18. The base calls binary was converted into FASTQ utilizing illumina package bcl2fastq v1.8.4. Adapters were trimmed away from the reads. Scythe v0.991 BETA and Sickle programs were used to remove adapter sequences. If the reads were shorter than 36 bp, those reads were dropped to produce cleaned data. FASTQ files were aligned against hg19 using Burrows-Wheeler Aligner, and the result files were converted to pileup format by samtools. Mutation candidates were determined according to the following criteria: (1) VAF of ≥0.5%; (2) minimum supporting reads of 5 at a variant position; (3) base quality and mapping quality of ≥20; (4) a minimum read depth of 1000; (5) if a variant was not consistent between paired-end reads, both reads were discarded. After mutation candidates calling, candidates were filtered based on the sequence data of WBC gDNA samples. Mean frequency of candidate single nucleotide variant (SNV) in WBC samples was considered as error rate. Each SNV allele frequency in cfDNA was compared with the error rate by one-sided binomial test, and if the p-value was higher than 0.05, we discarded the SNV. Additionally, we examined whether there was a strand bias at candidate SNV positions. We compared the genotypes inferred from the positive strand with those from negative strand using the chi-square test. If the p-value was lower than 0.05, the SNV was discarded. We further filtered mutation candidates by setting the cut-off VAF of 1.0%. However, for known hot spots (L702H, W742C, W742L, H875Y and T878A), variants with VAF of 0.5–1.0% were regarded as ‘true mutations’ if the SNVs were subsequently validated by dPCR.

### The functional analysis of V716M with a 3D permutation method

To interpret a functional importance of the rare mutation V716M, which is not considered a hot spot in *AR*, we estimated the location of V716M in an AR 3D protein structure (PDB id; 2PIX) with a 3D permutation method^27,28^. We calculated the average distance between hot spots and V716M. We then randomly selected amino acid residues from the AR 3D protein structure and calculated the average distance. This procedure was repeated 10,000 times, and a null distribution was generated. The p-value of the average distance of the mutations was evaluated using the null distribution.

### Prostate cancer cell lines

VCaP, LNCaP, 22rv1 and MDA PCa 2b (positive controls for *AR* amplification, T878A, H875Y and L702H, respectively) were purchased from American Type Culture Collection (ATCC, Manassas, VA, USA). DNA from each cell line was used as a positive control for the development of *AR* CN and mutations analysis. All experiment using cell lines were performed in triplicate.

### Mouse xenografts

To confirm successful detection of *AR* CN and mutations in cfDNA by dPCR, we analyzed plasma cfDNA from mice implanted with LNCaP or VCaP. A total of 1.0 × 10^7^ cells were injected subcutaneously into the bilateral flanks of 6 week-old male BALB/cA Jcl nude (nu/nu) mice (CLEA, Tokyo, Japan) under anesthetization. 1.0 ml blood was collected in an EDTA-containing tube, and 2 step centrifugation was performed within 2 h. cfDNA was extracted using the QIAamp DNA Blood Mini Kit (Qiagen). All experiments involving laboratory animals were performed in accordance with the Kyoto University Guidelines for Animal Experiments and approved by the Animal Research Committee at Kyoto University Graduate School of Medicine.

### Statistical analysis

cfDNA concentration between patients with CRPC and healthy males was compared using the Mann-Whitney U test. To evaluate the sensitivity of dPCR, *AR* CN between diluted VCaP gDNA and LNCaP gDNA, and the *AR* T878A mutation rate between diluted LNCaP gDNA and gDNA from healthy males were compared using the Student’s t-test. Frequency of *AR* amplification or mutations in cfDNA between samples collected at disease progression and while under response to treatment were compared using the chi-square test. Clinicopathologic factors associated with *AR* aberrations in cfDNA at disease progression were analyzed by univariate analysis (Fisher’s exact test for categorical variables or logistic regression for continuous variables). PSA-PFS rates after starting abiratetone and enzalutamide were estimated using the Kaplan-Meier method and the differences between groups were compared using the log-rank test and the Wilcoxon test. Univariate and multivariable Cox proportional hazard tests of PSA-PFS after starting abiraterone and enzalutamide were also performed. Differences were considered significant when a p-value < 0.05 was obtained. All statistical analyses were performed using JMP software version 13.0.0 for Windows (SAS institute Japan, Tokyo, Japan).

## Supplementary information

supplementary information
