# Supplementary material for: Clinical utility of androgen receptor gene aberrations in circulating cell-free DNA as a biomarker for treatment of castration-resistant prostate cancer
Source: Sci Rep. 2019 Mar 11;9:4030. doi: 10.1038/s41598-019-40719-y (PMC6411952; doi:10.1038/s41598-019-40719-y)
Supplement: Supplementary file 1 — supplementary information [file 41598_2019_40719_MOESM1_ESM.docx]

**Supplementary Information for**

**Clinical utility of androgen receptor gene aberrations in circulating cell-free DNA as a biomarker for treatment of castration-resistant prostate cancer.**

Takayuki Sumiyoshi^1^, Kei Mizuno^1,2^, Toshinari Yamasaki^1^, Yu Miyazaki^1^, Yuki Makino^1^, Kosuke Okasho^1^, Li Xin^1^, Noriaki Utsunomiya^1^, Takayuki Goto^1^, Takashi Kobayashi^1^, Naoki Terada^3^, Takahiro Inoue^1^, Tomomi Kamba^4^, Akihiro Fujimoto^2^, Osamu Ogawa^1^, and Shusuke Akamatsu^1*^

Correspondence should be addressed to S.A (E-mail: akamats@kuhp.kyoto-u.ac.jp).

**Supplementary information includes:**

Figures S1 to S7

Table S1 to S6

**Supplementary Figure S1**


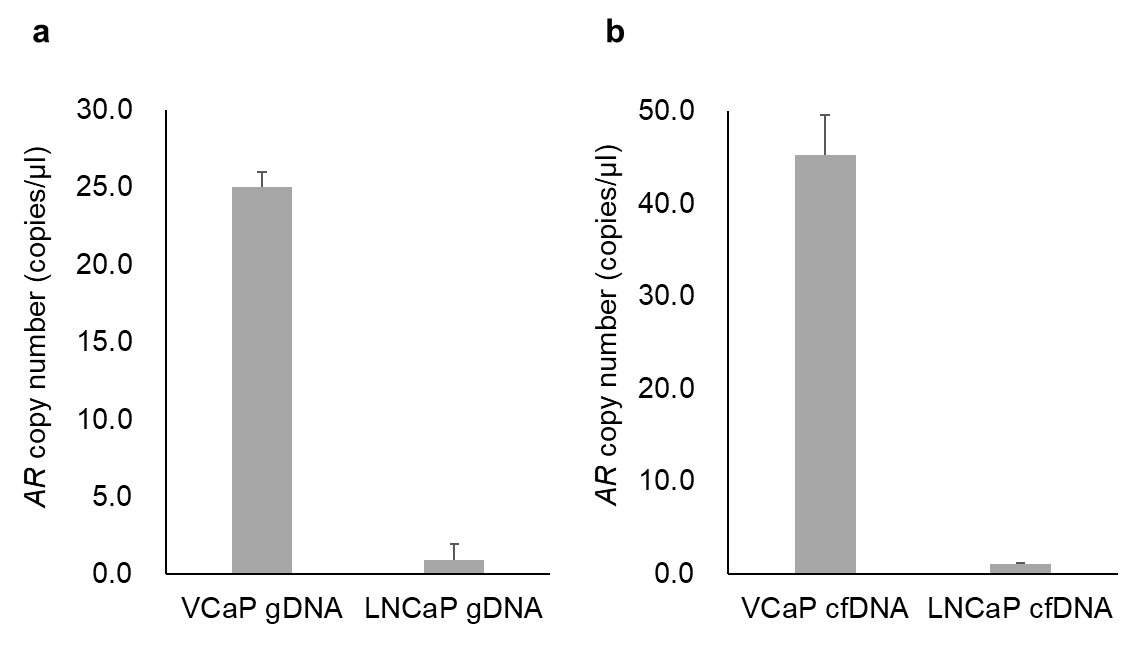


**Supplementary Figure S1.** ***AR* copy number (CN) analysis in DNA from cell lines by digital PCR.** (a) *AR* CN in VCaP gDNA and LNCaP gDNA were 25.02 copies/µl and 0.94 copies/µl, respectively. (b) *AR* CN in plasma cfDNA from mice implanted with VCaP (VCaP cfDNA) and LNCaP (LNCaP cfDNA) were 45.22 copies/µl and 1.07 copies/µl, respectively. The experiments were performed in triplicate, and error bars indicate standard deviation.

**Supplementary Figure S2**


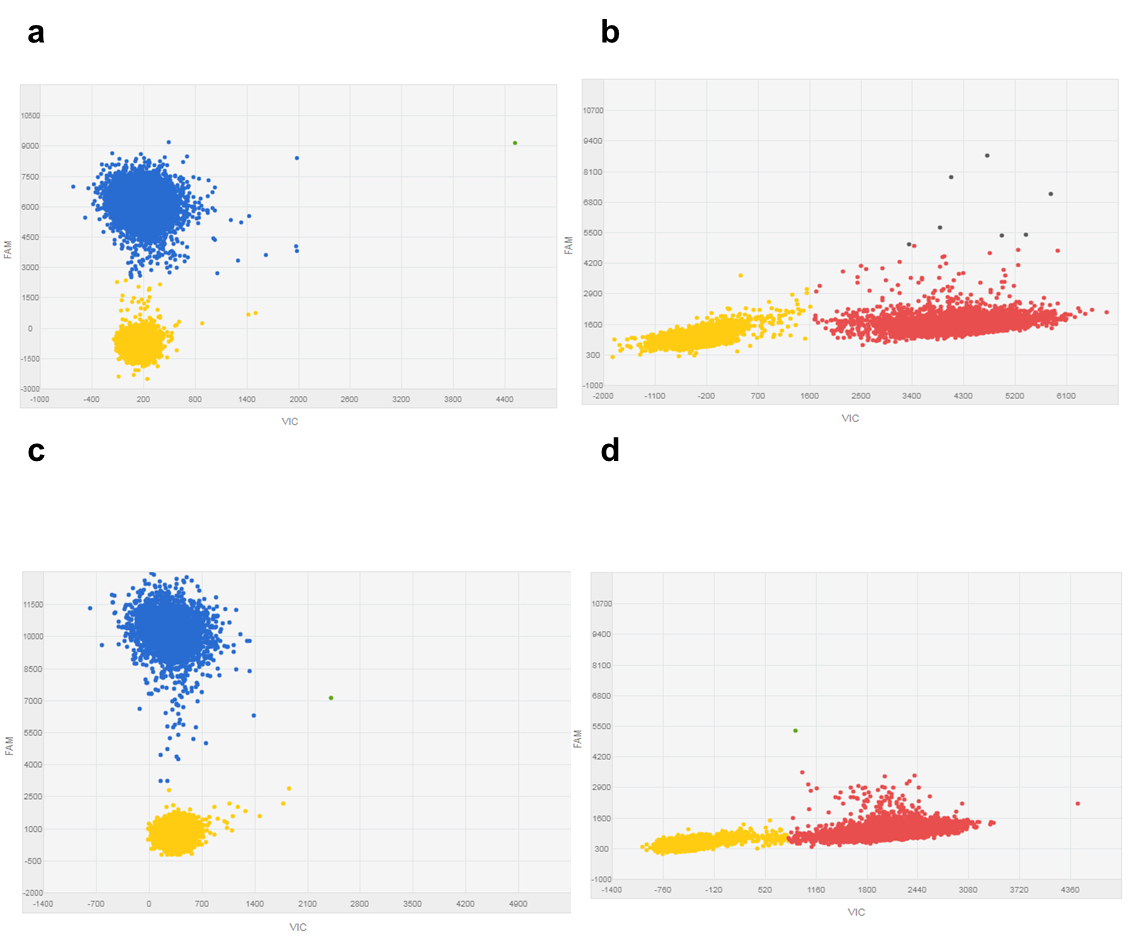


**Supplementary Figure S2. *AR* mutation analysis in DNA from cell lines by digital PCR.** Scatter plots of digital PCR analysis for mutation detection using LNCaP gDNA (a), VCaP gDNA (b) and cfDNA from mice implanted with LNCaP (c) and VCaP (d). The blue dots show positive droplets for the *AR* T878A mutation. The red dots show positive droplets for the *AR* wild type. The yellow dots show empty droplets. The experiments were performed in triplicate and representative scatter plots are presented.

**Supplementary Figure S3**

**
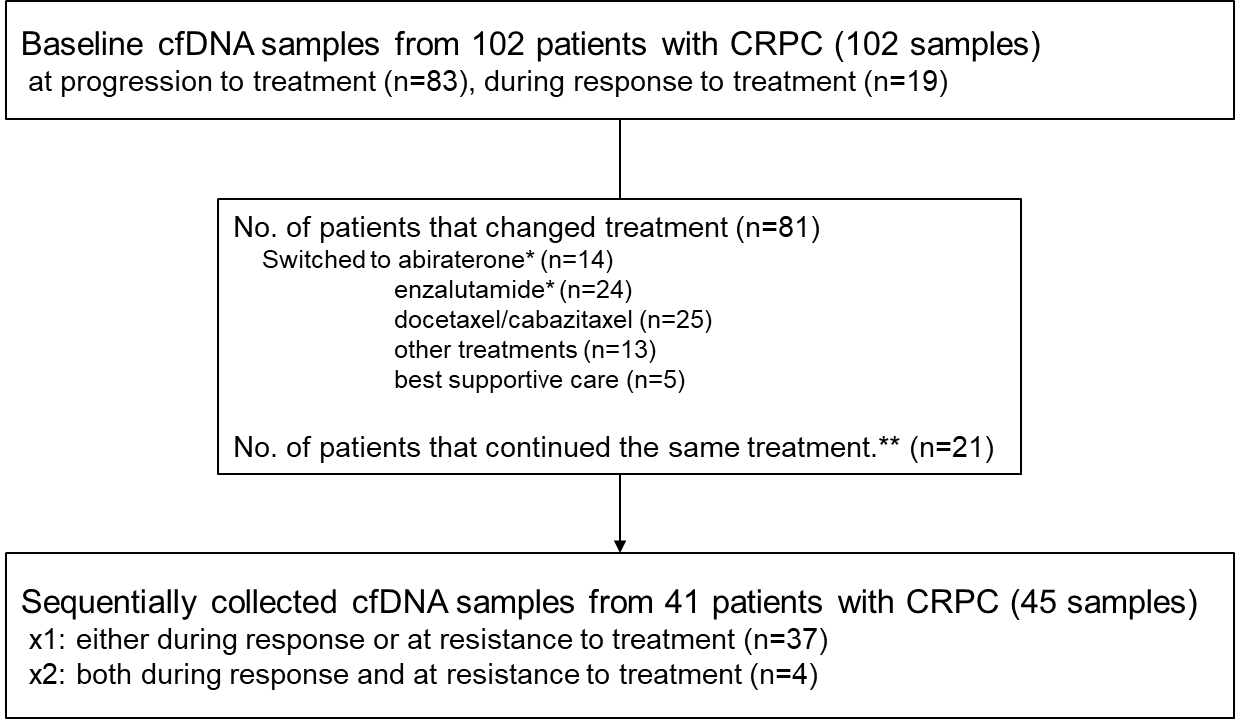
**

**Supplementary Figure S3. A scheme representing the time at which cfDNA samples were collected.**

*The cases include those who changed treatment because of adverse events to prior treatments (2 cases in abiraterone group and 1 case in enzalutamide group). ** Of the 21 patients that received same treatment after baseline cfDNA collection, 16 and 5 had cfDNA samples collected during response or at resistance to treatment, respectively.

**Supplementary Figure S4**

**
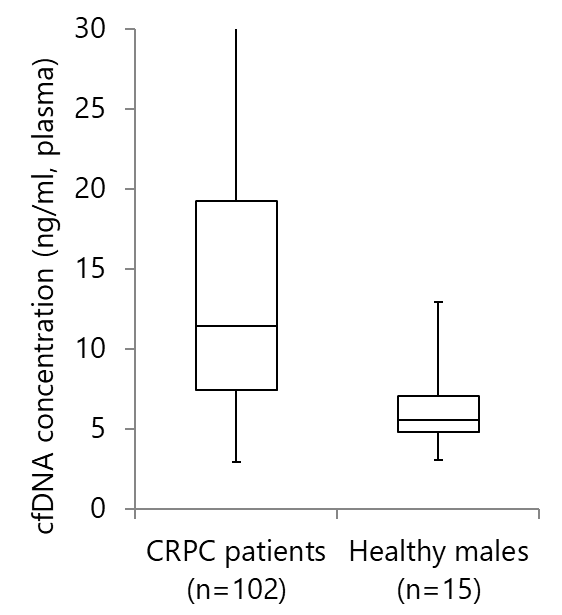
**

**Supplementary Figure S4. Median cfDNA concentration from patients with CRPC (n = 102) and healthy males (n = 15).** Box plots showing the cfDNA concentration in patients with CRPC and in healthy males. The cfDNA concentration from patients with CRPC was significantly higher than that from healthy males by the Mann-Whitney U test (median 11.36 ng/ml versus 5.52 ng/ml, p < 0.01). Data are expressed as median and interquartile range (box).

**Supplementary Figure S5**

**
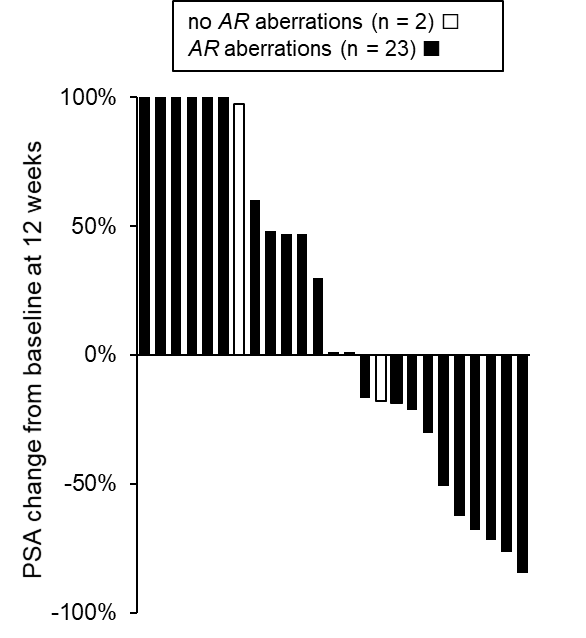
**

**Supplementary Figure S5. Waterfall plot of %PSA change from baseline at 12 weeks stratified by *AR* status for docetaxel and cabazitaxel.**

**Supplementary Figure S6**

**
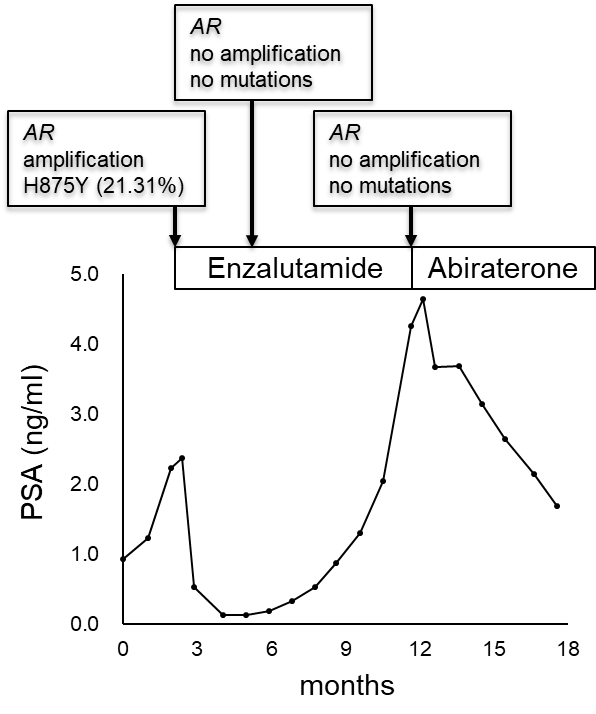
**

**Supplementary Figure S6. A representative case showing the feasibility of sequential analysis of cfDNA.** In patient KU-055, *AR* amplification and H875Y at baseline disappeared at response and did not re-emerge upon exhibiting resistance to enzalutamide. The patient subsequently responded to abiraterone.

**Supplementary Figure S7**

**
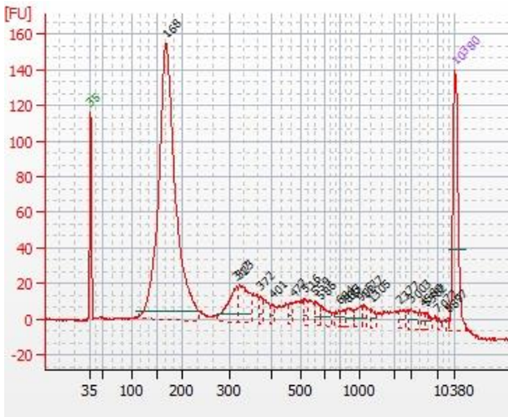
**

**Supplementary Figure S7. Size distribution of DNA extracted from plasma by the Bioanalyzer 2100.** The DNA peak at 168 bp was consistent with that of cfDNA. Y-axis shows fluorescent intensity proportional to DNA concentration. X-axis shows the size of DNA.

**Supplementary Table S1**

| Sample | Base change | Total  read count | Mutation candidate  read count | Variant allele frequency (%) |
| --- | --- | --- | --- | --- |
| LNCaP 0.5% | ChX: 66937326 G>A | 17674 | 146 | 0.83 |
|  | ChX: 66942821 C>T | 1279 | 8 | 0.63 |
|  | ChX: 66943548 G>A | 21272 | 140 | 0.66 |
| LNCaP 1.0% | ChX: 66931280 A>G | 15129 | 80 | 0.53 |
|  | ChX: 66943532 C>T | 21307 | 110 | 0.52 |
|  | ChX: 66943532 C>T | 16501 | 90 | 0.55 |
|  | ChX: 66943543 C>T | 25529 | 130 | 0.51 |
| LNCaP 5.0% | ChX: 66931444 G>A | 22523 | 228 | 1.01 |
|  | ChX: 66931528 C>T | 23189 | 166 | 0.72 |
|  | ChX: 66943532 C>T | 17832 | 92 | 0.52 |
|  | ChX: 66937326 G>A | 15401 | 92 | 0.60 |
| LNCaP 10% | ChX: 66943532 C>T | 26866 | 204 | 0.76 |
|  | ChX: 66943540 C>T | 26868 | 236 | 0.88 |
| LNCaP 100% | ChX: 66943542 G>A | 28519 | 180 | 0.63 |

**Supplementary Table S1. Mutation candidates in the range of 0.5% to 1.0% other than T878A in LNCaP gDNA.**

**Supplementary Table S2**

| sample | Base change | Total read count | Mutation candidate read count | Variant allele frequency (%) |
| --- | --- | --- | --- | --- |
| control08 | ChX: 66943635 C>A | 35498 | 206 | 0.58 |
| control08 | ChX: 66943532 C>T* | 9826 | 52 | 0.53 |
| control12 | ChX: 66942787 C>A | 8637 | 44 | 0.51 |
| control15 | ChX: 66943540 C>T* | 16774 | 92 | 0.55 |

**Supplementary Table S2. Mutation candidates in the range of 0.5% to 1.0% in cfDNA from healthy males.** *The candidates were also detected in LNCaP gDNA.

**Supplementary Table S3**

| Sample | Tissue site | *AR* amplification (copy number, copies/μl) | | *AR* mutation (variant allele frequency, %) | |
| --- | --- | --- | --- | --- | --- |
|  |  | cfDNA | tissue gDNA | cfDNA | tissue gDNA |
| KU-012 | prostate | No (1.37) | No (1.36) | No | No |
| KU-032 | prostate | No (1.37) | No (1.11) | No | No |
| KU-033 | prostate | Yes (5.86) | Yes (2.08) | No | No |
| KU-069 | prostate | Yes (11.30) | Yes (2.28) | No | No |
| KU-071 | prostate | Yes (2.62) | Yes (2.16) | No | No |
| KU-089 | prostate | Yes (1.76) | Yes (1.85) | No | No |
| KU-015 | prostate | No (1.37) | Yes (5.78) | No | No |
| KU-074 | prostate | No (1.26) | Yes (2.98) | No | No |
| KU-102 | prostate | No (1.11) | Yes (25.55) | No | No |
| KU-116 | prostate | No (1.50) | Yes (2.51) | No | No |
| KU-112 | prostate | No (1.51) | Yes (7.75) | W742L (0.52) | No |
| KU-029 | bone | Yes (1.88) | Yes (1.64) | W742C (20.49) T878A (24.59) | W742C (51.20) T878A (58.77) V904A (1.92) |

**Supplementary Table S3. Comparison of *AR* status for cfDNA and gDNA from matched tumor tissue.**

**Supplementary Table S4**

| **Supplementary Table S4.** Univariate analysis (Fisher's exact test for categorical variables or logistic regression for continuous variables) examining the association between baseline clinicopathological factors and *AR* status in cfDNA collected at disease progression from patients with CRPC (n=83). | | | | | |
| --- | --- | --- | --- | --- | --- |
| Category | | n | *AR* aberrations in cfDNA | | *P* |
|  |  |  | n | % |  |
| PSA |  |  |  |  | 0.673 |
| Gleason score* | 8-10 | 15 | 10 | 66.7 | 1.000 |
|  | 6-7 | 65 | 43 | 66.2 |  |
| Visceral metastasis | yes | 17 | 13 | 76.5 | 0.563 |
|  | no | 66 | 43 | 65.2 |  |
| ECOG PS | ≥2 | 26 | 18 | 69.2 | 1.000 |
|  | 0-1 | 57 | 38 | 66.7 |  |
| Hemoglobin | ≤LLN | 16 | 16 | 100 | 0.001 |
|  | ≥LLN | 67 | 40 | 59.7 |  |
| ALP | ≥360 | 29 | 22 | 75.9 | 0.326 |
|  | ≤359 | 54 | 34 | 63 |  |
| LDH | ≥227 | 28 | 22 | 78.6 | 0.144 |
|  | ≤226 | 55 | 34 | 61.8 |  |
| Treatment immediately prior to baseline sample collection | hormone therapy | 75 | 49 | 65.3 | 0.264 |
|  | chemotherapy | 8 | 7 | 87.5 |  |
| No. of prior anti-androgen/ chemotherapy | ≥3 | 50 | 40 | 80.0 | 0.004 |
|  | ≤2 | 33 | 16 | 48.5 |  |
| Bicalutamide resistance | yes | 80 | 55 | 68.8 | 0.246 |
|  | no | 3 | 1 | 33.3 |  |
| Flutamide resistance | yes | 56 | 41 | 73.2 | 0.136 |
|  | no | 27 | 15 | 55.6 |  |
| Estramustine phosphate resistance | yes | 32 | 27 | 84.4 | 0.015 |
|  | no | 51 | 29 | 56.9 |  |
| Abiraterone resistance | yes | 29 | 25 | 86.2 | 0.008 |
|  | no | 54 | 31 | 57.4 |  |
| Enzalutamide resistance | yes | 30 | 26 | 86.7 | 0.007 |
|  | no | 53 | 30 | 56.6 |  |
| Docetaxel resistance | yes | 24 | 21 | 87.5 | 0.019 |
|  | no | 59 | 35 | 59.3 |  |
| Cabazitaxel resistance | yes | 4 | 4 | 100 | 0.299 |
|  | no | 79 | 52 | 65.8 |  |
| cfDNA concentration |  |  |  |  | 0.018 |
| Abbreviations: PSA, prostate-specific antigen; ECOG PS, Eastern Cooperative Group Performance Status; ALP, Alkaline Phosphatase; LDH, Lactate dehydrogenase; LLN, Lower Limit of Normal. | | | | | |

**Supplementary Table S4.**　**Univariate analysis (Fisher's exact test for categorical variables or logistic regression for continuous variables) examining the association between　baseline clinicopathological factors and *AR* status in cfDNA collected at disease progression from patients with CRPC (n = 83).** *The Gleason score of three patients remained unknown.

**Supplementary Table S5**

| Category | | n | Median  PSA-PFS (months) | Unvariate analysis | | Multivariable analysis | |
| --- | --- | --- | --- | --- | --- | --- | --- |
|  |  |  |  | HR (95% CI) | *P* | HR (95% CI) | *P* |
| PSA |  |  |  | 1.0 (0.99-1.03) | 0.363 |  |  |
| Gleason score* | 8-10 | 12 | 230.5 |  | 0.266 |  |  |
|  | 6-7 | 1 | not reached |  |  |  |  |
| Visceral metastasis | Yes | 3 | 70 | 2.05 (0.29-9.61) | 0.422 |  |  |
|  | No | 11 | 342 | - |  |  |  |
| ECOG PS | ≥2 | 3 | 70 | 2.72 (0.55-11.37) | 0.203 |  |  |
|  | 0-1 | 11 | not reached | - |  |  |  |
| Hemoglobin | <LLN | 0 | - |  |  |  |  |
|  | ≥LLN | 14 | 230.5 |  |  |  |  |
| ALP | ≥360 | 4 | 342 | 0.78 (0.11-3.43) | 0.764 |  |  |
|  | ≤359 | 9 | 101.5 | - |  |  |  |
| LDH | ≥227 | 0 | - |  |  |  |  |
|  | ≤226 | 14 | 230.5 |  |  |  |  |
| No. of prior anti-androgen/ chemotherapy | ≥3 | 7 | 70 | 5.04 (1.14-34.76) | 0.032 | 1.78 (0.24-15.56) | 0.567 |
|  | ≤2 | 7 | 342 | - |  | - |  |
| cfDNA concentration |  |  |  | 1.26 (1.00-1.62) | 0.048 | 1.57 (1.11-2.63) | 0.009 |
| *AR* status in cfDNA | *AR* amplification, L702H, H875Y or T878A | 8 | 66.5 | 3.74 (0.85-25.79) | 0.083 | 16.77 (2.02-333.1) | 0.007 |
|  | without *AR* amplification, L702H, H875Y and T878A | 6 | 342 | - |  | - |  |

**Supplementary Table S5.** **Univariate and multivariable Cox proportional hazard test examining the association between baseline clinicopathological factors and PSA progression free survival rate in patients starting abiraterone after collection of cfDNA (n=14).** *The Gleason score of one patient remained unknown.

**Supplementary Table S6**

| Category | | n | Median  PSA-PFS (months) | Unvariate analysis | | Multivariable analysis | |
| --- | --- | --- | --- | --- | --- | --- | --- |
|  |  |  |  | HR (95% CI) | *P* | HR (95% CI) | *P* |
| PSA |  |  |  | 1.003  (0.999-1.007) | 0.054 | 1.00 (1.00-1.01) | 0.363 |
| Gleason score* | 8-10 | 18 | 335 | .46 (0.10-2.34) | 0.325 |  |  |
|  | 6-7 | 5 | 127 | - |  |  |  |
| Visceral metastasis | yes | 3 | 112 | 3.87 (0.20-26.45) | 0.296 |  |  |
|  | no | 21 | 292 | - |  |  |  |
| ECOG PS | ≥2 | 8 | 112 | 6.78 (1.64-33.38) | 0.009 | 6.74 (1.14-38.39) | 0.037 |
|  | 0-1 | 16 | 335 | - |  | - |  |
| Hemoglobin | <LLN | 4 | - | 5.15 (0.23-56.30) | 0.242 |  |  |
|  | ≥LLN | 20 | 292 | - |  |  |  |
| ALP | ≥360 | 7 | 335 | 0.93 (0.20-3.40) | 0.916 |  |  |
|  | ≤359 | 17 | 292 | - |  |  |  |
| LDH | ≥227 | 6 | 292 | 2.04 (0.43-7.80) | 0.339 |  |  |
|  | ≤226 | 18 | 335 | - |  |  |  |
| No. of prior anti-androgen/ chemotherapy | ≥3 | 13 | 292 | 1.31 (0.37-5.13) | 0.677 |  |  |
|  | ≤2 | 11 | not reached | - |  |  |  |
| cfDNA concentration |  |  |  | 1.00 (1.00-1.00) | 0.222 |  |  |
| *AR* status in cfDNA | *AR* amplification | 11 | 204 | 2.84 (0.78-13.29) | 0.115 |  |  |
|  | no *AR* amplification | 13 | not reached | - |  |  |  |

**Supplementary Table S6.** **Univariate and multivariable Cox proportional hazard test examining the association between baseline clinicopathological factors and PSA progression free survival rate in patients starting enzalutamide after collection of cfDNA (n=24).** *The Gleason score of one patient remained unknown.
